# Functional Study of the Chitinase *CaChi93* Gene from the Mycoparasitic *Cladosporium* sp. SYC23

**DOI:** 10.3390/jof12040237

**Published:** 2026-03-26

**Authors:** Chen Chen, Mingjiao Li, Ruotian Gao, Mengling Yan, Ting Zhou, Yanping Tang, Jing Li

**Affiliations:** 1 College of Biological Science and Food Engineering, Southwest Forestry University, Kunming 650224, China; 2 Forest Resources Exploitation and Utilization Engineering Research Center for Grand Health of Yunnan Provincial Universities, Southwest Forestry University, Kunming 650224, China

**Keywords:** mycoparasitism, *Cladosporium* sp. SYC23, identification of gene, chitinase, enzyme activity assay, prokaryotic expression

## Abstract

To identify chitinase genes from the genome of the mycoparasitic *Cladosporium* sp. strain SYC23, bioinformatical analyses and real-time quantitative PCR (RT-qPCR) were employed to screen mycoparasitism-associated genes at 12, 24, 48, and 72 h post-induction with *Aecidium pourthiaea* rust spores. A total of eight chitinase genes were identified from SYC23 via bioinformatics analysis and designated *CaChi34*, *CaChi40*, *CaChi45*, *CaChi67*, *CaChi82*, *CaChi92*, *CaChi93*, and *CaChi286* based on sequence and phylogenetic analyses. Analysis of the chitinase protein sequence characteristics revealed molecular weights ranging from 33.86 to 286.03 kDa and theoretical isoelectric points from 4.48 to 7.7. All *CaChi* genes contained the conserved GH18 domain, and promoter analysis showed that each harbored MYB-binding sites and pathogen-responsive elements. Mycoparasitism-related sequence clustering analysis indicated that the chitinase sequences of SYC23 shared the closest phylogenetic relationship with those from *Trichoderma* sp. RT-qPCR results following rust spore induction showed that five *CaChi* genes reached their highest expression levels at 24 h post-induction, *CaChi45* was most highly expressed at 72 h post-induction, *CaChi93* was continuously upregulated, and *CaChi82* was continuously downregulated throughout the induction period. His-tagged recombinant CaChi93 protein was purified from *E. coli* and characterized. The results demonstrate that the enzymatic activity of CaChi93 was 0.929 U/mg, with optimal reaction conditions at 65 °C and pH 7. Treatment of *A. pourthiaea* rust spores with the recombinant CaChi93 chitinase confirmed that CaChi93 could effectively dissolve rust spore walls. In conclusion, this study confirms that the mycoparasitic *Cladosporium* sp. strain SYC23 can secrete chitinase to degrade the rust spore wall and induce spore death, thereby providing novel gene resources and a theoretical basis for the biological control of *A. pourthiaea*.

## 1. Introduction

*Photinia Lindl.* comprises evergreen trees, shrubs, and small arborescent taxa belonging to the genus *Photinia* within the family Rosaceae (order Rosales) [1]. Globally, the genus encompasses approximately 140 species, mainly distributed across eastern and southern Asia. In China, around 60 documented species are primarily distributed in Sichuan, Yunnan, Guizhou, Fujian, Guangdong, and other provincial regions. As a prominent subtropical tree genus in southern China, *Photinia* is highly valued for both landscaping applications and timber production [2,3]. Furthermore, members of the genus display remarkable biological activities, including analgesic, antipyretic, antitumor, herbicidal, insecticidal, and fungicidal properties [4]. Rust disease is a widespread and destructive disease affecting *Photinia* species, frequently causing chlorosis, deformation, and swelling in leaves, axillary buds, and shoots, which severely impairs plant growth, development, and ornamental value. *A. pourthiaea* is identified as the causal agent of rust disease in *Photinia prionophylla*. This pathogen mainly infects young trees and seedlings under three years old, inducing necrosis of leaves, axillary buds, and shoots, thereby drastically compromising the normal growth and ornamental application of *Photinia* plants [5]. Rust diseases constitute a category of plant pathogens causing severe damage to agricultural, forestry, and ornamental crops. The economic losses caused by plant diseases such as *P. prionophylla* rust [6,7], pear rust [8,9], wheat stripe rust [10], and coffee rust [11,12] are extensive. The control of rust disease of *Photinia* remains a significant challenge, and mycoparasitic fungi possessing fungal parasitism capabilities are crucial biocontrol agents for suppressing rust disease growth. They represent a class of micobial resources with considerable potential for biocontrol. The most typical examples are genera *Trichoderma* sp. [13], *Coniothyrium* sp. [14], *Cladosporium* sp. [15,16], and *Pestalotiopsis* sp. [17,18]. Currently, research on the biological control of plant pathogens using parasitic fungus *Trichoderma* is the most advanced and comprehensive. From crude toxin extraction to omics studies and gene function validation [19], it has been discovered that *Trichoderma* sp. primarily achieves biological control by parasitizing plant pathogens through two mechanisms: toxin production and the secretion of spore wall-degrading enzymes.

Chitinase (EC 3.2.1.14) is a class of specific glycosyl hydrolases (G H) that catalyzes the hydrolysis of natural polymers such as chitin, chitosan, and peptidoglycan. It is also one of the main components of the cell wall degradation enzymes in mycoparasitic fungi, which can catalyze the degradation of chitin in the cell walls of plant pathogens [20]. Chitinase is widely distributed in various organisms, including animals, plants, fungi, and bacteria, with fungi being one of the primary sources of this enzyme [21,22]. Fungal chitinase mainly belongs to the GH18 family and exhibits diverse physiological functions, including morphogenesis, autolysis, nutrient uptake, and fungal mycoparasitism [20,23]. Chitin is one of the main components of fungal cell walls and typically works in conjunction with other polysaccharides (such as glucans and mannans) to maintain the integrity and functionality of the cell wall.

However, the specific function of chitinase during the mycoparasitic process of *Cladosporium* sp. strain SYC23 against the rust pathogen *A. pourthieae* has not yet been systematically elucidated. Accordingly, this study focused on the identification and characterization of the chitinase gene family in the mycoparasitic strain SYC23 using bioinformatics approaches, with the ultimate goal of exploring its potential role in degrading the cell walls of *A. pourthiaea* rust spores. We examined chitinase activity at different stages during rust spore induction, investigated the expression patterns of chitinase-related genes associated with mycoparasitism using real-time fluorescence quantitative PCR (RT-qPCR), and analysed the physiological activity of mycoparasitism-related chitinases by expression in *Escherichia coli*. The research findings provide insight into the role of chitinase genes in the mycoparasitism process of *Cladosporium* sp., and into the biosafety control mechanisms for *P. prionophylla* rust disease.

## 2. Materials and Methods

### 2.1. Strain Cultivation

Spores from the rust fungus *A. pourthieae*, growing on the plant *P. prionophylla*, were used to induce gene expression in *Cladosporium* sp. strain SYC23. The mycoparasitic fungus *Cladosporium* sp. strain SYC23 was isolated from rust spore masses of *P. prionophylla* [6]. Specifically, disinfected rust-infected *P. prionophylla* leaves and their adjacent tissues were precisely excised into small fragments (approximately 0.5 cm × 0.5 cm) using a sterile scalpel. These tissue fragments were subsequently placed uniformly on potato dextrose agar (PDA) medium plates (retained as provided), with 3–4 fragments evenly distributed per plate. All plates were incubated at a constant temperature of 25 °C. On the third day of incubation, meticulous observations were performed, and a small aliquot of mycelium was sampled from the margin of newly emerged mycelial growth. This mycelial sample was transferred to a fresh PDA medium plate under strict sterile conditions, and this purification process was repeated 2–3 times to obtain axenic colonies, thereby guaranteeing the accuracy and reliability of subsequent experimental procedures. Finally, small sections of these pure colonies were excised and transferred to test tubes containing PDA slants for long-term preservation and subsequent experimental use. The SYC23 strain, which had been activated for 7 days, was inoculated into potato dextrose broth (PDB) medium and cultured in a constant-temperature shaker at 28 °C and 150 r/min for 48 h. Seven-day activated cultures of strain SYC23 were inoculated into liquid potato dextrose broth (PDB) and cultured at 28 °C with shaking at 150 r/min for 48 h. After the collection of *Aecidium pourthiaea* spores, the rust spores were repeatedly frozen and thawed at −20 °C, then ground with a mortar and pestle, and the ground rust spores were collected and observed under a microscope, and then the rust spores were sterilized under the condition of 121 °C for 20 min, which were then used as rust spore walls of the inducers. Subsequently, inactivated rust spores were added to the culture medium at a ratio of 1% for induction, referring to the method described by Mei et al. [15]. Parallel treatments with 1% colloidal chitin and a blank control (without any rust spores) were set up, and all groups were subjected to induced cultivation for 0, 24, 48, and 72 h, respectively. The fermentation broths were then collected and filtered through four layers of lens paper under aseptic conditions to separate the mycelia from the supernatants. The harvested mycelia were immediately snap-frozen in liquid nitrogen for subsequent experiments. The resulting supernatants were sterilized by filtration through a 0.22 mm membrane filter to obtain crude enzyme solutions, which were stored at 4 °C for further use. (All reagents and chemical supplies used in this experiment were supplied by Shanghai Genomics, Shanghai, China).

### 2.2. Determination of N-Acetyl-D-Glucosamine Content

The N-acetyl-D-glucosamine (GlcNAc) content in the fermentation broth of strain SYC23 under various treatments was quantified using the 3,5-dinitrosalicylic acid (DNS) method [24,25,26]. A volume of 1 mL crude enzyme solution was added to a reaction mixture consisting of 1 mL of 1% colloidal chitin and 2 mL of phosphate buffer (pH 7.0) that had been preheated to 50 °C. The mixture was incubated in a thermostatic water bath at 50 °C for 1 h. Following cooling in an ice bath, 3 mL of DNS reagent was added. The mixture was incubated in a boiling water bath at 100 °C for 10 min, cooled to room temperature, and then diluted to a final volume of 10 mL. After centrifugation, the absorbance of the supernatant at 540 nm (OD_540_) was determined.

### 2.3. Identification and Bioinformatics Analysis of Hypothetical Chitinase Amino Acid Sequence in SYC23

The raw genome sequencing data of strain SYC23 have been deposited in the NCBI database under the BioProject accession number PRJNA1175971. Experimental procedures were performed according to the methods described by Junges et al. [27] and Sui Wenjing et al. [28] for chitinase gene mining. We first performed gene mining using assembled data and annotated genomic data, employing the GH18 family conserved domains (S/AxGG and DxxDxDxE) as templates. The nucleotide sequences of chitinases from Trichoderma parareesei were retrieved from the NCBI database. These sequences were subsequently aligned against the fungal genome of strain SYC23 using local BLAST analysis. The combined and filtered hits were used to define the putative chitinase gene family in SYC23. The identified candidate chitinase genes were further subjected to prediction and functional analysis using various online bioinformatics tools. The detailed software programs and their corresponding functions are listed in Appendix A. Known chitinase gene sequences from *Trichoderma* sp., *Sclerotinia* sp., and *Pestalotiopsis* sp. were retrieved from the NCBI database and aligned with the putative chitinase family genes from strain SYC23 for phylogenetic tree construction. Multiple sequence alignment was performed using the MAFFT version 7 online server. A neighbor-joining phylogenetic tree was subsequently constructed using MEGA 11.0 software with the bootstrap method (1000 replicates), and the resulting tree was visualized and optimized using the ChiPlot (https://www.chiplot.online/) online platform.

### 2.4. Analysis of Chitinase Gene Expression in SYC23 Strain

Total RNA was extracted from *Cladosporium* sp. SYC23 at various time points after rust spore induction using TRIzol reagent according to the manufacturer’s instructions, followed by DNase treatment. RNA concentration and purity were determined using a NanoDrop 2000 spectrophotometer (Thermo Fisher Scientific, Waltham, MA, USA). RNA integrity was verified by 1% agarose gel electrophoresis. First-strand cDNA was synthesized using the SynScript^®^ III RT SuperMix for qPCR kit, and the resulting cDNA was used as the template for subsequent qPCR analysis. The qPCR reaction mixture (20 μL total volume) contained 10 μL ArtiCanCEO SYBR qPCR Mix, 0.8 μL forward primer, 0.8 μL reverse primer, and 1 μL cDNA template. Amplification was performed on an ABI QuantStudio StepOne Plus real-time quantitative PCR system using the following program: 95 °C for 5 min (1 cycle) and 95 °C for 15 s, 60 °C for 20 s, 72 °C for 20 s (40 cycles), with fluorescence signal acquisition at 72 °C. The ITS gene of *Cladosporium* sp. SYC23 was used as the internal reference gene. Primers were designed using Primer Premier 5 software (Table 1) and synthesized by Qingke Bioengineering Co., Ltd. (Beijing, China). Experiments were repeated three times. Relative quantification was calculated using the 2^−ΔΔCt^ method [29], and data were analyzed by one-way ANOVA using SPASS 26.0 software.

### 2.5. Construction of the pET28a-CaChi93 Expression Vector

The CaChi93 gene was cloned into the pET-28a(+) vector, which introduces a 6× His-tag at the N-terminus of the recombinant protein. Nickel affinity chromatography (Ni-NTA resin) was used for purification because the 6× His-tag specifically binds to Ni^2+^ions, allowing for one-step purification of the recombinant protein with high purity and yield. Using the cDNA sequence of the chitinase gene *CaChi93* from strain SYC23 as a template, the N-terminal signal peptide and C-terminal transmembrane domains of *CaChi93* were removed. The target gene sequence, which contained the conserved domains and functional regions, was then chemically synthesized by Shanghai Sangon Biotech Co., Ltd. The target protein sequence was ligated into the *pET-28a*(*+*) expression vector using NdeI and XhoI double digestion. The recombinant plasmid *pET28a-CaChi93* was confirmed by double digestion and subsequently transformed into *Escherichia coli Rosetta (DE3)* competent cells. Following heat shock at 42 °C, the cells were spread on Luria–Bertani (LB) agar plates supplemented with 30 μg/mL kanamycin and 34 μg/mL chloramphenicol, then incubated at 37 °C. Single colonies were selected for PCR verification. Plasmid DNA was extracted simultaneously using the SanPrep Column Plasmid Mini Prep Kit (Sangon, Shanghai, China) and subjected to unidirectional sequencing of the target fragment at Sangon Biotech Co., Ltd. Sequence alignment verified the accuracy of the constructed sequence, and the positive recombinant strain *pET28a*(*+*)*-CaChi93/E. coli Rosetta* (*DE3*) was successfully obtained.

### 2.6. Expression and Purification of the Recombinant Chitinase CaChi93 Protein

The recombinant strain *pET28a*(*+*)*-CaChi93/Escherichia coli Rosetta* (*DE3*) was spread on LA agar plates supplemented with 30 μg/mL kanamycin and 34 μg/mL chloramphenicol, followed by incubation at 37 °C for 24 h. A single colony was then inoculated into LB liquid medium containing the same antibiotics and cultured at 37 °C with shaking. When the optical density at 600 nm (OD_600_) reached 0.6, IPTG was added to a final concentration of 0.5 mM to induce protein expression. Two induction conditions were applied: overnight incubation at 20 °C, and incubation at 37 °C for 6 h. A non-induced culture was used as the negative control.

The cultured cells were harvested by centrifugation at 4000 rpm for 10 min using a refrigerated centrifuge, and the supernatant was discarded to collect the cell pellet. The harvested cells were resuspended in 30 mL of lysis buffer and lysed using an ultrasonic homogenizer under the following conditions: power 140 W, 5 s sonication followed by 5 s pause, with a total processing time of 30 min. Cell debris was removed by centrifugation at 8000 r/min and 4 °C for 15 min. The supernatant containing crude protein was collected and further purified by Ni-column affinity chromatography. The recombinant *pET28a*(*+*)*-CaChi93* protein was detected by 12% sodium dodecyl sulfate-polyacrylamide gel electrophoresis (SDS-PAGE) and stained with 1% Coomassie Brilliant Blue R-250. The purified protein fraction was dialyzed against protein storage buffer (50 mM Tris, 300 mM NaCl, 0.1% sarkosyl, 2 mM DTT, pH 8.0), concentrated, filtered for sterilization, aliquoted into 1 mL tubes, and stored at −80 °C. The identity of the purified protein was further verified by Western blot analysis. A mouse anti-6×His tag monoclonal antibody (Sangon Biotech, Shanghai, China) was used as the primary antibody, followed by the corresponding secondary antibody.

### 2.7. Study on the Enzymatic Activity Characteristics of Recombinant Chitinase CaChi93

Following the methods of Li [30], Qiao and Cheng [31] with minor modifications, chitinase activity was determined using the DNS method. A 20 μL aliquot of diluted recombinant chitinase was mixed with 100 μL of 1% colloidal chitin prepared in phosphate buffer (pH 7.0), and the mixture was incubated at 50 °C for 30 min. Then, 90 μL of DNS reagent was added, and the mixture was boiled for 5 min for color development. After cooling, the absorbance at 540 nm (OD_540_) was measured using a spectrophotometer. A blank control was prepared using inactivated recombinant chitinase under the same reaction conditions. All reactions were performed in triplicate. One unit (U) of enzyme activity was defined as the amount of enzyme required to release 1.0 μmol of N-acetylglucosamine per minute under the described conditions.

$$U=\frac{m\times 10^{3}}{n\times t\times 221}$$

*U*—enzyme activity (U/mg); *m*—mass of N-acetylglucosamine (g) determined from the standard curve; *n*—amount of enzyme used in the reaction (mg); *t*—enzyme reaction time (min).

### 2.8. Study on the Inactivation of Aecidium pourthiaea Rust Spores by Recombinant Chitinase CaChi93

To evaluate the disruptive effect of CaChi93 chitinase on rust spores, 100 μL of reconstituted CaChi93 chitinase was added to sterile 1.5 mL centrifuge tubes. A total of 0.01 g of rust spores was inoculated into each tube to ensure sufficient contact and uniform distribution between the spores and enzyme solution. The enzyme-treated spores were incubated for 0 h, 24 h, 48 h, 72 h, 96 h, and 7 d, with sterile water used as the control. All treatments were performed in three biological replicates. After incubation, samples were collected, stained with 0.1% trypan blue for 5 min, and observed under an optical microscope to evaluate the degree of rust spore damage.

## 3. Results

### 3.1. Identification and Sequence Characterization of the CaChis Genes

The chitinase gene family in the genome of *Cladosporium* sp. SYC23 was identified using Hidden Markov Models (HMM) combined with genome functional annotation. Conserved domains of the initially screened candidates were further verified using the NCBI Conserved Domain Database (NCBI-CDD) and SMART database. Finally, eight chitinase genes designated as *CaChis* were identified, all belonging to the glycoside hydrolase family 18 (GH18). These genes were named according to their predicted molecular weights, as listed in Table 2. As summarized in Table 2, the eight *CaChis* genes encoded proteins ranging from 316 to 2633 amino acids (aa), with predicted molecular weights of 33.86–286.03 kDa and theoretical isoelectric points (pI) of 4.48–7.7. Three chitinase genes contained putative signal peptide motifs. Subcellular localization predictions revealed that four gene products (proteins) were predicted to be extracellular, possibly acting as secreted enzymes or signaling molecules. *CaChi40* was predicted to localize to the mitochondria, whereas *CaChi92* and *CaChi93* were cytoplasmic proteins. Transmembrane domains were detected in both *CaChi286* and *CaChi93*. Collectively, these results reveal substantial divergence in the physicochemical properties of the *CaChis* genes family.

### 3.2. Phylogenetic Analysis, Structural Features, and Analysis of Conserved Domains of CaCHis

**Figure 1:**
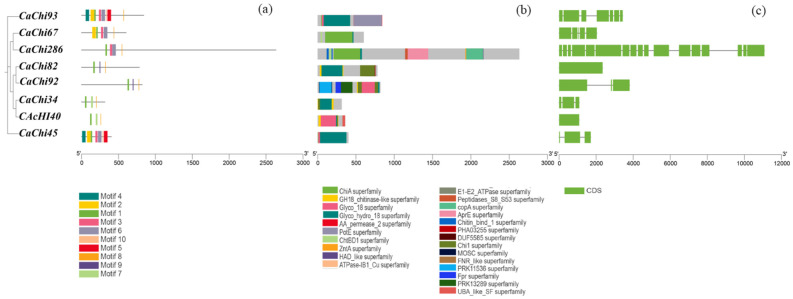
Schematic diagram of conserved motifs, conserved domains, and gene structures of the *CaChis* genes. (**a**) Conserved motifs; (**b**) Conserved domains; (**c**) Gene structures.

The structural domain prediction results for the *CaChi* genes are presented in Figure 1. All eight chitinase family genes contain a complete glycoside hydrolase family 18 (Glyco_hydro_18) catalytic domain, which is the core functional unit of GH18 family chitinases. Additionally, these genes collectively harbor 18 distinct structural domains, indicative of the significant structural diversity within the chitinase gene family of strain SYC23. Furthermore, all eight chitinase protein sequences contain the conserved catalytic domain motif DxxDxDxE (Motif 1), which is predominantly located at the C-terminal end of the proteins. Only *CaChi92* possesses a catalytic domain at the N-terminal end. Among these genes, only *CaChi93*, *CaChi67*, and *CaChi45* harbor the substrate-binding domain motif S/AxGG (motif 2). Among the SYC23 genes, three possess only one exon, while the remaining genes contain between two and seventeen exons. Notably, *CaChi286* contains over seventeen exons, with a sequence length of 2633 amino acids—approximately eight times that of the shortest sequence, *CaChi34*. This demonstrates the complex structural specificity of the *CaChis* genes.

**Figure 2:**
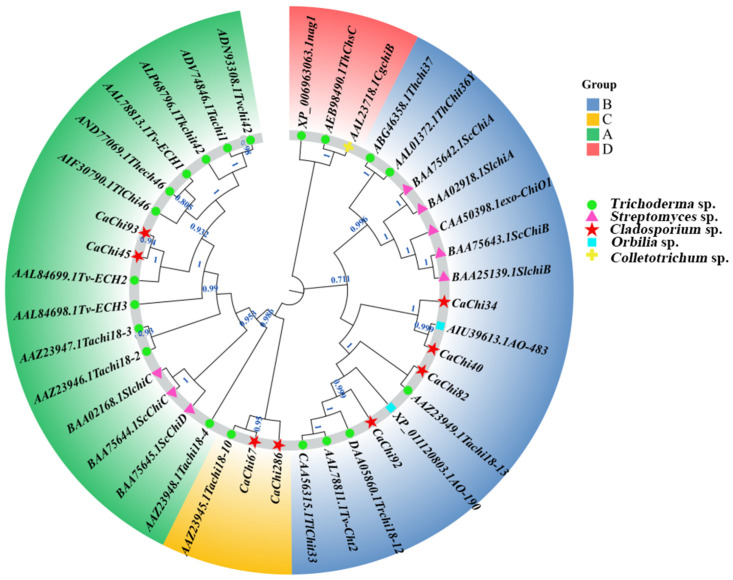
Phylogenetic tree of chitinase family members from *Trichoderma* sp., *Sclerotinia* sp., *Pestalotiopsis* sp., and *Cladosporium* sp.

**Figure 3:**
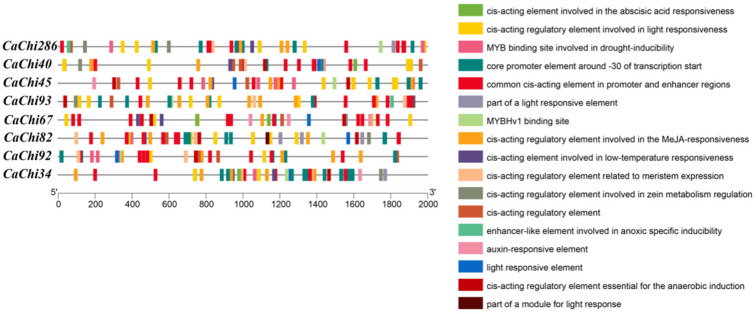
Diagram of *cis*-acting elements of the promoter sequences of the *CaChis*.

**Figure 4:**
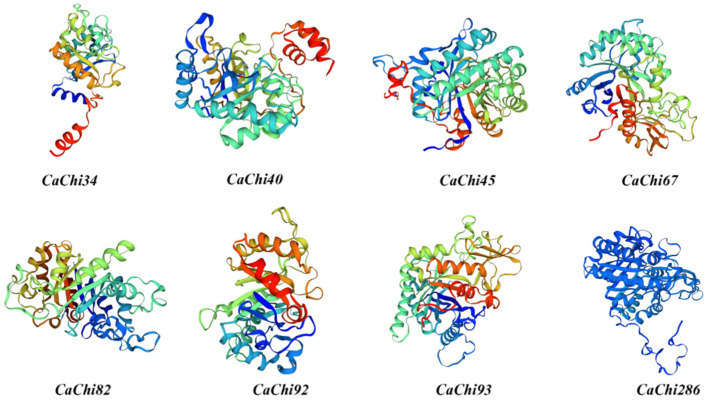
Prediction of tertiary structure of CaChis proteins.

**Figure 5:**
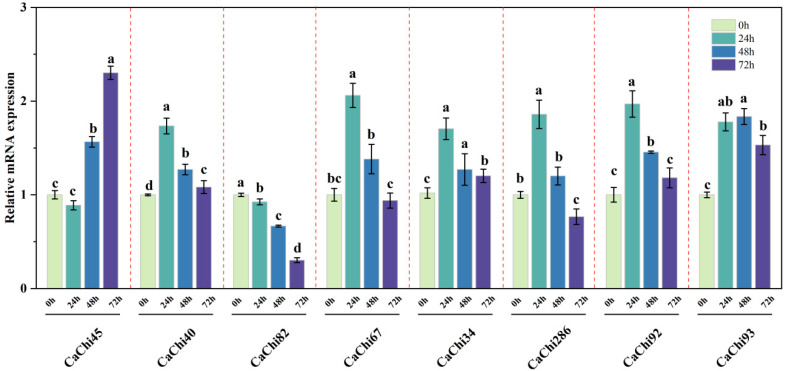
Relative expression of chitinase genes induced by *Aecidium pourthiaea* spores. (a, b, c and d are significance symbols used to indicate whether there is a statistically significant difference in gene expression levels between different time points (0 h/24 h/48 h/72 h); the same letter indicates no significant difference between groups, whilst different letters indicate a significant difference (*p* < 0.05)).

**Figure 6:**
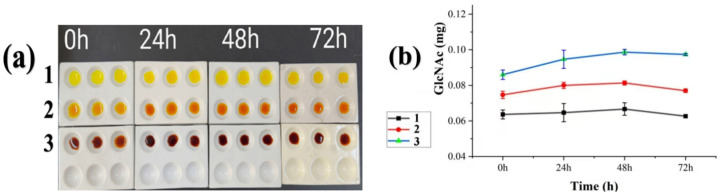
Changes in reducing sugars in fermentation broth of strain SYC23 under different culture conditions. (**a**): DNS color reaction; (**b**): Changes in N-acetylglucosamine content. Note: (1) Blank control; (2) 1% colloidal chitosan treatment; (3) *Pyronium* rust spore treatment.

**Figure 7:**
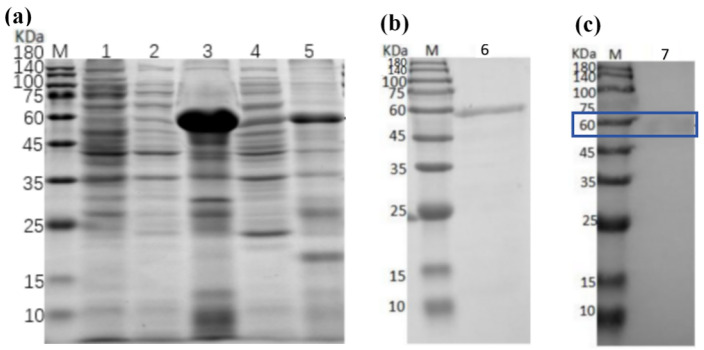
SDS-PAGE and Western-blot of recombinant CaChi93. (**a**) Recombinant protein expression analysis in (**a**). (**b**) Purified recombinant protein in (**b**). (**c**) Western-blot results of supernatant induced at 20 °C in (**c**). M: protein marker; 1: uninduced sample; 2: supernatant induced at 20 °C; 3: precipitation induced at 20 °C; 4: supernatant induced at 37 °C; 5: precipitation induced at 37 °C; 6&7: final purified protein.

**Figure 8:**
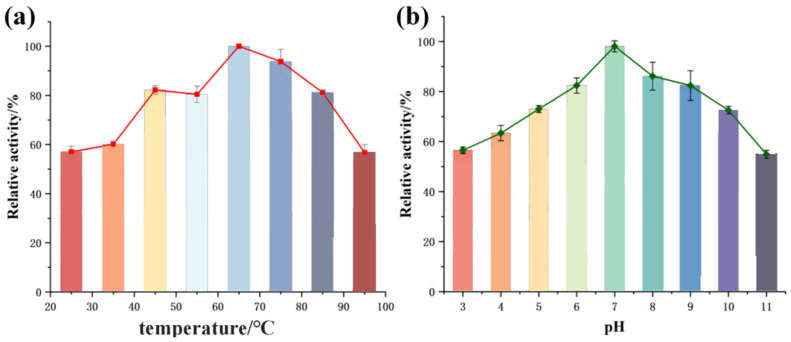
Determination of optimum temperature and pH of CaChi93 protein. (**a**) Relative enzyme activities of CaChi93 at various temperatures under pH 7.0. (**b**) The relative enzyme activity of CaChi93 at each pH at 50 °C.

**Figure 9:**
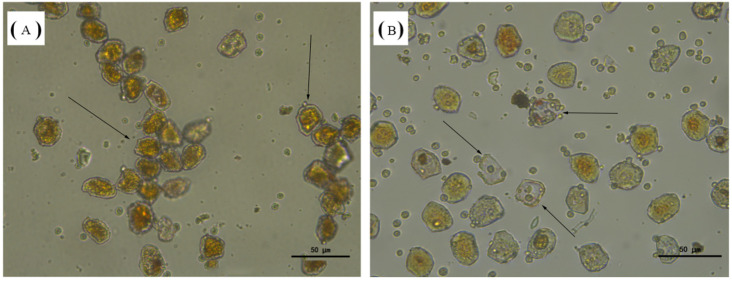
Morphological observation of rust treated with the recombinant CaChi93 protein. (**A**): Water treatment 7 d (400 times); (**B**): enzyme treatment 7 d (400 times).

Figure 2 shows the phylogenetic tree of chitinase family genes. Results indicate that by downloading published chitinase gene sequences from mycoparasitic fungi and performing cluster analysis with the chitinase sequence from strain SYC23, and referencing the classification by Verena et al. [32] and Goughenour [33], the chitinase family genes were classified into four groups: A, B, C, and D (Figure 2, Figure 3, Figure 4, Figure 5, Figure 6, Figure 7, Figure 8 and Figure 9). Among these, the chitinase genes *CaChi45* and *CaChi93* from strain SYC23 were classified into Group A. They share the closest phylogenetic relationship with the gene Tv-ECH2 (AAL84699.1), both belonging to the endochitinase category. Most genes in Group A are *Trichoderma* chitinase genes involved in the bioparasitism process [34]. Four genes (*CaChi34, CaChi40, CaChi82, and CaChi92*) were grouped into cluster B. Specifically, *CaChi40* clustered with the AO-483 (AIU39613.1) gene, while *CaChi82* clustered with the Tachi18-13 (AAZ23949.1) gene and *CaChi286* and *CaChi67* clustered with the *Trichoderma* Tachi18-10 (AAZ23945.1) gene, forming Group C.

### 3.3. Analysis of the CaChis Family Gene Promoters

Figure 3 shows the *cis*-acting element predictions for the 2000 bp upstream region of *CaChis*, which contains numerous light-responsive elements, hormone-responsive elements, MYB-related binding sites, pathogen response elements, and *cis*-acting elements involved in defense responses. This indicates that both environmental stress and pathogen infection may influence chitinase gene expression, and MYB transcription factors may also participate in regulating this process.

### 3.4. Construction and Analysis of CaChis Protein Structures

Secondary structure prediction for the CaChis sequences was conducted using the SOPMA online server, and the corresponding results are presented in Table 3. The secondary structures of the CaChis proteins are mainly composed of α-helices, β-sheets, random coils and extended strands, with respective proportions ranging from 16.24% to 39.24%, 2.3% to 8.19%, 11.39% to 19.95%, and 39.07% to 68.16%. The secondary structure components varied significantly among the eight sequences. Further analysis using the SWISS-MODEL online platform predicted the tertiary structure of *CaChis* proteins (as shown in Figure 4), with results consistent with the secondary structure analysis.

### 3.5. Expression Relative to the CaChis Genes

To explore the role of chitinase family genes in the rust spores of *Aecidium pourthiaea* that are mycoparasitized by SYC23, real-time fluorescent RT-qPCR was used to detect the expression levels of eight *CaChis* genes in SYC23 rust fungi at different stages following rust spore induction (Figure 5). Results showed that among rust spore-induced genes, *CaChi34*, *CaChi40*, *CaChi67*, *CaChi286*, and *CaChi92* exhibited peak relative expression at 24 h, followed by a declining trend. *CaChi45* initially decreased at 24 h but then rose continuously, reaching approximately 2.4-fold relative expression at 72 h compared to 0 h. The *CaChi93* gene exhibited high expression at all three time points post-rust spore induction, showing extremely significant relative expression differences compared to 0 h. The relative expression of the *CaChi82* gene exhibited a continuous decline. In summary, *CaChis* genes showed significant correlation with rust spore induction.

### 3.6. Changes in N-Acetylglucosamine Production by SYC23 Strain Under Different Conditions

Figure 6a presents the colorimetric reaction of the culture broth of strain SYC23 with 3,5-dinitrosalicylic acid (DNS) under different treatment conditions. N-acetyl-D-glucosamine (GlcNAc), the hydrolysis product of colloidal chitin by chitinase, reacts with DNS to form a characteristic orange-brown chromogen; the intensified color in the rust spore-induced group indicated that strain SYC23 secreted chitinase with robust enzymatic activity under this induction condition. Figure 6b demonstrates that the GlcNAc content in the culture broth of SYC23 induced by *Photinia prionophylla* rust spore walls was consistently higher than that in both the blank control and 1% colloidal chitin treatment groups across all time points. Moreover, the GlcNAc content in the rust spore wall-induced group reached its maximum at 24 h post-induction. Combined with the chitinase gene expression profiling results (Figure 5), these findings confirm that *Aecidium pourthiaea* rust spores effectively induce chitinase synthesis in *Cladosporium* sp. SYC23, with the most prominent induction effect on chitinase activity observed at 24 h post-induction.

### 3.7. Protein Expression Assays and Purification

The recombinant strain (DE3) was cultured under two distinct conditions: overnight incubation at 20 °C, and continuous culture at 37 °C for 6 h. After sonication, the supernatant and pellet were collected and analyzed by sodium dodecyl sulfate-polyacrylamide gel electrophoresis (SDS-PAGE), as shown in Figure 7a. Results indicated that the optimal conditions for CaChi93 protein expression in *E. coli* were 15 °C overnight induction, with the protein predominantly distributed in the pellet. The findings confirmed successful expression of a protein with an approximate molecular weight of 56.8 kDa, consistent with expectations (Figure 7b). The purified protein was subjected to Western blot analysis to detect recombinant CaChi93 protein expression (Figure 7c; the blue box indicates the expression of protein Cachi93).

### 3.8. Chitinase Activity and Optimal Temperature and pH

The standard curve for N-acetyl-D-glucosamine measured using an enzyme-linked immunosorbent assay reader yielded an R^2^ value of 0.9918, with the equation y = 0.01816x − 0.14693. Under conditions of pH 7.0, 1% colloidal chitosan concentration, and incubation at 65 °C for 30 min, the recombinant CaChi93 enzyme activity was measured at 0.292 U/mg.

As depicted in Figure 8a, the impact of various temperatures on the enzymatic activity of the recombinant CaChi93 chitinase was investigated. The relative enzymatic activity of CaChi93 chitinase initially increased and subsequently decreased with elevating temperature, reaching a maximum at 65 °C. Above 70 °C, the relative activity declined gradually, reaching its lowest value (approximately 57%) at 95 °C. Figure 8b presents the relative enzymatic activity of the recombinant CaChi93 chitinase across a gradient of pH values. At pH 3, the enzyme exhibited a relative activity of approximately 67%, with activity elevating progressively as the pH increased and peaking at pH 7. With a further increase in alkalinity, the relative activity of the recombinant CaChi93 declined gradually, falling to a minimum of about 55% at pH 11. Collectively, these results confirm that the recombinant CaChi93 chitinase retains over 50% of its relative enzymatic activity over a broad temperature range of 25–95 °C and under strongly acidic to alkaline pH conditions, thus exhibiting robust thermostability and pH tolerance. The enzyme achieved maximal activity at 65 °C and pH 7.0.

### 3.9. Enzyme Activity on Aeciospores

Microscopic observations of *Aecidium pourthiaea* rust spores treated with the purified recombinant CaChi93 chitinase are presented in Figure 9. Comparative analysis revealed that *Aecidium pourthiaea* rust spores in the experimental group were generally more dispersed, accompanied by abundant small aggregates around the spores. With prolonged incubation time, the number of extracellular aggregates gradually increased, the intracellular contents became paler, and the spore cell walls progressively thinned. After 48 h of enzyme treatment, the rust spore walls displayed obvious deformation and shrinkage, with orange-yellow intracellular contents. In contrast, *Aecidium pourthiaea* rust spores in the control group began to agglomerate at 24 h, and the degree of agglomeration intensified over time. At 7 days post-treatment, rust spores in the experimental group became permeable, showing significantly faded intracellular contents, numerous empty spore shells, and the most severe cell wall rupture (Figure 9B). Collectively, these results demonstrate that the recombinant CaChi93 chitinase possesses strong degrading activity against the cell walls of *Aecidium pourthiaea* rust spores.

## 4. Discussion

In this study, bioinformatics approaches were employed to mine and analyze GH18 family genes in the mycoparasitic fungus strain SYC23, and eight GH18 family chitinase genes were identified. Promoter prediction was subsequently performed using the PlantCare online tool, which revealed that these gene promoters were abundant in cis-acting elements associated with pathogen responses, plant hormone signaling, and light responses. Previous studies have demonstrated that several key elements, including the W-box, as-1, G-box, CGTCA-motif, ABRE, TGACG-motif, and O2-site, are involved in fungal stress responses and environmental signal transduction [35,36,37,38,39]. Furthermore, the upstream promoter regions of *CaChis* genes all contain MYB recognition and binding sites, suggesting that GH18 family genes may interact with MYB transcription factors in regulating defense gene expression and environmental stress responses.

Mycoparasitism is a sophisticated biological process in which chitinases catalyze the hydrolysis of β-1,4-glycosidic linkages in chitin—the primary structural component of fungal cell walls—completely degrading it into N-acetyl-β-D-glucosamine. This enzymatic breakdown disrupts and solubilizes the cell wall integrity of phytopathogenic fungi, thereby compromising their structural and functional viability. Chitinases are therefore recognized as key hydrolases mediating mycoparasitic interactions, and they play a pivotal role in the mycoparasitic suppression of phytopathogens as well as the biological control of plant fungal diseases. [40]. Previous studies have demonstrated that cell walls derived from host fungi can act as effective inducers to activate the expression of genes associated with mycoparasitic recognition [41]. Two induction systems have been commonly adopted, including solid-state culture [40,42] and liquid culture [40,43]. Therefore, spores from the rust fungus *Aecidium pourthieae*, growing on the plant *Photinia prionophylla*, were used to induce gene expression in *Cladosporium* sp. strain SYC23. Mycelia of *Cladosporium* sp. SYC23 harvested at 0, 24, 48, and 72 h post-induction with rust spore walls were used as experimental materials. Real-time quantitative PCR (RT-qPCR) and the 3,5-dinitrosalicylic acid (DNS) assay were jointly performed to verify the inductive effect of holly rust spore walls on chitinase biosynthesis in this strain. The results revealed that seven out of the eight *CaChi* genes were significantly upregulated at 24 h post-induction with rust spore walls, while only *CaChi82* displayed a continuous downregulation trend throughout the 0–72 h induction period. These results demonstrate that the 0–24 h period serves as the key induction phase for chitinase gene expression in SYC23, suggesting that the chitinase genes of this strain may play critical roles in the early stage of its mycoparasitic interaction with rust fungi. The DNS method [44] operates on the principle that 3,5-dinitrosalicylic acid undergoes a colorimetric reaction with N-acetyl-β-D-glucose derived from hydrolyzed chitin. The reducing sugar content was determined by measuring the optical density at 540 nm (OD_540_) of the reaction mixture using colorimetry. Under the assay conditions, the concentration of N-acetyl-β-D-glucosamine was directly proportional to the color intensity of the reaction solution. As presented in Figure 6a, the culture broth of strain SYC23 induced by *Aecidium pourthiaea* rust spores displayed a distinct orange-brown color, while no obvious color change was observed in the control group. This result indicates that strain SYC23 secreted a high level of chitinase upon initial exposure to *Aecidium pourthiaea* rust spores. The *Escherichia coli* prokaryotic expression system enables efficient, convenient, and rapid synthesis of target gene products, thus providing a reliable basis for the functional characterization of genes [45]. As was reported by Yan [46], when expressing the endochitinase T6-Echi18-5 from *Trichoderma longibrachiatum* in *E. coli* cells, a 60 kDa protein was isolated from the cell lysate precipitate after 6 h of IPTG induction at both 28 °C and 37 °C. Li [26] conducted prokaryotic expression analysis of the chitinase *CS1801* from *Streptomyces amylomuconastrum*, yielding a protein of 45 kDa in size and determining its enzymatic activity to be 0.132 U/mL. The recombinant CaChi93 chitinase in this study exhibited an enzymatic activity of 0.292 U/mg, with colloidal chitin serving as the optimal substrate for enzymatic hydrolysis. This activity is comparable to that of the recombinant CS1801 chitinase from *Streptomyces amylosus*. Reports indicate that bacterial chitinases exhibit a broad optimal pH range, whereas fungal and actinomycete chitinases predominantly have acidic optimal pH values. For instance, the chitinase Chi2375 from the thermotolerant microorganism *Microbulbifer thermotolerans* YLW106 has an optimal pH of 8.0 and an optimal temperature of 55 °C [47]. Studies on recombinant protease activity of chitinases (VC1073, VCA0027, and VC1952) from the marine microorganism *Vibrio cholerae* revealed acidic optimal pH values of 6, 6, and 5, respectively, with all exhibiting high activity and stability across a broad pH range [48]. The optimal pH for AnChiB chitinase from *Aspergillus niger* is 5 [49]. Recombinant chitinases Xn-Chi60 and Xn-Chi70, cloned from *Bacillus thuringiensis*, both exhibit an optimal pH of 6 [50]. Similarly, the recombinant chitinase chi8 from *Trichoderma guizhouense* strain NJAU4742 also exhibits an optimal pH of 6 [51]. Furthermore, the optimal temperatures of these recombinant chitinases are relatively similar, ranging from 50 °C to 60 °C. In this study, the recombinant chitinase derived from *Cladosporium* sp. exhibited an optimal pH of 7, retained stable enzymatic activity over a wide pH range, and displayed strong pH tolerance. This resembles the optimal pH of the recombinant CS1801 chitinase from *Streptomyces amyloliquefaciens* [26], but differs from the optimal pH of common fungal and actinomycete chitinases, indicating that the recombinant CaChi93 chitinase from *Cladosporium* sp. SYC23 exhibits strong pH stability. Furthermore, the optimal temperatures for enzyme activity vary significantly among chitinases from different fungal sources. For example, the recombinant CS1801 chitinase from *Streptomyces amyloliquefaciens* displays maximal activity at 50 °C, whereas the recombinant endochitinase Chi8 from *Trichoderma guizhouense* exhibits optimal activity at 30–40 °C. In contrast, the recombinant CaChi93 chitinase in this study exhibits optimal activity at 65 °C, with relatively stable enzyme activity maintained within the 25–95 °C range, indicating good thermal stability of the recombinant CaChi93 chitinase. Research indicates that chitinase plays a crucial role in the antagonistic effects of *Trichoderma* against plant pathogens such as *Fusarium* and *Fusarium* head blight [41,52]. For instance, the recombinant expression of *Trichoderma harzianum* chitinase gene 02524 exhibits potent inhibitory effects on the growth of plant pathogens such as *Fusarium oxysporum*, *Phytophthora cinnamomi*, and *Sclerotinia sclerotiorum* [53]. Harighi et al. (2007) demonstrated that purified chitinase-42 (Chi42) from *Trichoderma atroviride* cleaved the cell wall of *R. solani* and inhibited mycelial growth [54]. Furthermore, recombinant CaChi93 chitinase was successfully expressed in *E. coli* and shown to effectively inactivate *Aecidium pourthiaea* spores. This study further confirmed that the recombinant CaChi93 chitinase can increase the permeability and reduce the thickness of rust spore cell walls, ultimately leading to cell wall rupture and leakage of intracellular contents. These observations demonstrate that the recombinant CaChi93 chitinase derived from *Cladosporium* sp. SYC23 possesses the capacity to destroy rust spores from infected *P.prionophylla* plants. Collectively, these findings suggest that the chitinase produced by strain SYC23 may exert its biological control function during mycoparasitism by penetrating and degrading the cell walls of *Aecidium pourthiaea* rust spores.

## 5. Conclusions

This study demonstrates that the branch-spore fungus SYC23 possesses biocontrol potential by producing chitinase to destroy *Aecidium pourthiaea* rust spores. The mycoparasitic *Cladosporium* sp. SYC23 possesses eight chitinase genes, exhibiting significant differential expression upon rust spore wall induction. Furthermore, the CaChi93 chitinase was successfully recombinantly expressed in *Escherichia coli*, and its significant destructive effect on *Photinia prionophylla* rust spore walls was confirmed through antibacterial activity assays.

## Figures and Tables

**Table 1:** Relative Quantitative Primers.

Primer Name	Primer Sequence (5′ to 3′)	Primer Name	Primer Sequence (5′ to 3′)
ITS1-F	TCCGTAGGTGAACCTGCGG	ITS4-R	TCCTCCGCTTATTGATATGC
CaChi45-F	TTCGCCGTTCCTATGTCCAC	CaChi45-R	ACCTTCCTTTGGGCTCTGTG
CaChi40-F	CGGTATGGGTGTACCTCAGC	CaChi40-R	ACTATTGAGAGCGGCGACAG
CaChi82-F	GCGTGTCGAACAACAACACT	CaChi82-R	AATCTCCGTCTTGCGACCTG
CaChi67-F	GGGTCTACAAGTTTGCGTGC	CaChi67-R	GCATCCGCTTGGTAGAGACA
CaChi34-F	CCTGGATGTGGGTGAAGGAG	CaChi34-R	GCGGATCAGATGTCGTTCCA
CaChi286-F	CATCAGACTCGACACCCTCG	CaChi286-R	GAATCTCACCCTCGCCAGTC
CaChi92-F	TATGATGTGGGATGCGAGCC	CaChi92-R	CACCCTCACACCATCCCTTC
CaChi93-F	CATGGTTGATGTCACGGCTG	CaChi93-R	TTTGCCTGCTACTGCTGAGG

**Table 2:** Basic physical and chemical properties of *CaChis*.

Gene Sequence ID	Gene Name	Length (aa)	Molecular Weight (kDa)	pI	Aliphatic	Hydrophilicity Index (GRAVY)	Signal Peptide	Subcellular Localization	Transmembrane Structure	Structure Domain
NODE_9_0000379_t	*CaChi45*	403	44.91	5.31	67.59	−0.465	-	extr:	-	Glyco_hydro_18
NODE_11_0000661_t	*CaChi40*	361	39.51	5.79	84.29	−0.212	-	mito:	-	Glyco_hydro_18, UBA_like_SF
NODE_32_0002076_t	*CaChi82*	782	82.34	7.7	63.82	−0.218	+	extr:	-	Glyco_hydro_18
NODE_34_0002517_t	*CaChi67*	603	66.73	4.74	65.56	−0.341	-	cyto:	-	Glyco_hydro_18, ChtBD1
NODE_39_0002890_t	*CaChi34*	316	33.86	4.48	81.2	−0.142	-	extr:	-	Glyco_hydro_18
NODE_44_0003251_t	*CaChi286*	2633	286.03	4.94	69.85	−0.317	+	extr:	5	Glyco_hydro_18, ChtBD1, ZntA, AprE, Peptidases_S8_S53
NODE_53_0003630_t	*CaChi92*	821	91.54	5.86	74.57	−0.354	-	plas:	-	Glyco_hydro_18, MOSC, FNR_like
NODE_73_0003950_t	*CaChi93*	842	92.52	5.89	86.56	0.048	+	plas:	10	Glyco_hydro_18, GH18_chitinase, AA_permease_2, PotE

**Table 3:** CaChis Secondary structure prediction information.

Sequence ID	α-Helix/%	β-Angle/%	Irregular Curling/%	Extension Chain/%
CaChi34	39.24	5.38	43.99	11.39
CaChi40	30.47	3.6	52.08	13.85
CaChi45	30.02	5.64	50.12	14.39
CaChi67	20.73	3.15	60.53	15.59
CaChi82	16.24	2.3	68.16	13.3
CaChi92	28.62	6.09	47.5	17.78
CaChi93	32.78	8.19	39.07	19.95
CaChi286	22.86	3.68	59.13	14.32

## Data Availability

The sequencing data for this project are available via the National Center for Biotechnology Information (NCBI), the accession number is PRJNA1175971.

## Appendix A. Detailed List of Bioinformatics Analysis Online Websites

**Database**	**Function**	**Online Websites**
CD-search	Conservative Domain Prediction	https://www.ncbi.nlm.nih.gov/Structure/cdd/wrpsb.cgi (accessed on 16 March 2026)
SMART		https://smart.embl.de/ (accessed on 16 March 2026)
ExPASy-ProtParm	Prediction of Basic Physical and Chemical Properties	https://www.psort.org/psortb/ (accessed on 16 March 2026)
SignalP 5.0	Predictive signal peptide site	https://services.healthtech.dtu.dk/service.php?SignalP-5.0 (accessed on 16 March 2026)
TMHMM	Predicting Transmembrane Structures	https://services.healthtech.dtu.dk/service.php?TMHMM-2.0 (accessed on 16 March 2026)
MEME	Conservative Module Prediction	https://meme-suite.org/meme/tools/meme (accessed on 16 March 2026)
WoLF PSORT	Predicting Subcellular Localization	https://wolfpsort.hgc.jp/ (accessed on 16 March 2026)
Sompa	Predicting Secondary Structure	http://npsa-pbil.ibcp.fr/cgi-bin/npsa_automat.pl?pa-ge=npsa_sopma.html (accessed on 16 March 2026)
Plantcare	Prediction of Promoter *Cis*-Actin Elements	https://bioinformatics.psb.ugent.be/webtools/plantcare/html/ (accessed on 16 March 2026)
Protein blast	Chitinase Homologous Sequence Search	http://blast.ncbi.nlm.nih.gov/Blast.cgi (accessed on 16 March 2026)
MAFFT v.7.0	Multiple sequence alignment	https://mafft.cbrc.jp/alignment/software/ (accessed on 16 March 2026)
ChiPlot	Phylogenetic Tree construction and optimization	https://www.chiplot.online/ (accessed on 16 March 2026)
